# Aging Well: Toward a Way of Life for All People

**Published:** 2005-06-15

**Authors:** Barbara A Hawkins

**Affiliations:** Indiana University Center on Aging and Aged, Smith Research Center

The world's population as a whole is living longer. Population aging is a trend that will bring new social, political, and economic demands to countries throughout the world (1). Without proactive steps to create individual and environmental changes that promote aging well, the escalating costs of health and social support systems for an aging population will be unsustainable both domestically and worldwide (2).

The concept of aging well, which is based on a nonmedical approach to promoting health and well-being, is fundamental to increasing length and quality of life. Aging well promotes personal behaviors and life-course environments that limit functional declines, especially those caused by chronic conditions, to help older adults maintain their independence and health. Aging well emphasizes the idea that people can maintain satisfying and healthy lives as they age by exercising the choices that optimize healthy, active, and secure lives (1,3,4). Aging well is a dynamic, interactive process that creates long-term, positive change by involving individuals in the physical, social, economic, historical, and cultural contexts of their environments.

Development of an aging-well framework fits well with articulated health goals for the United States and the international community (5). While many countries have made significant gains in addressing national health objectives, a nonmedical framework for aging well has not yet been fully developed. This knowledge gap persists in spite of evidence linking one or more specific areas, such as social support, physical activity, and material security, to longevity and quality of life.

Although no single formula automatically results in aging well, research conducted by Fry and colleagues provides several illustrations of factors that contribute to aging well in different cultures (6). For example, in Hong Kong a comfortable old age is marked by family and social qualities that demonstrate open-mindedness and tolerance. In the United States, engagement, vitality, and activity are associated with aging well. As Fry and colleagues point out, "Having good health and comfortable pensions does not hurt either." Across the cultures studied, determinants that are important to aging well — including physical health, mental effectiveness, material security, social resources and relationships, and meaningful daily activity — are common, but their relative contributions may vary.

Research supports the concept of aging well (1,5,7-11). Current international research efforts are moving forward from identifying the determinants of aging well to testing an integrated model and investigating viable individual and environmental indices of aging well. These research initiatives will help to identify populations, environments, services, programs, and public policies that support the continued involvement of older adults in positive, productive, and healthy living.

One example of such an initiative grew out of the 21^st^-century research agenda on aging developed by the United Nations and the International Association of Gerontology. The agenda outlines research goals related to healthy aging, quality of life, and measurements of active aging (5). These research goals provided the original impetus and continuing direction for 25 researchers from 12 countries to convene in June 2000 at Indiana University to develop a multinational research collaboration. Originally known as the Global Ageing Initiative and renamed the Global Ageing Research Network (GARNet) in 2004, GARNet includes partners from Australia, Austria, the United Kingdom, Greece, Malta, Italy, Sweden, China, Netherlands, Luxembourg, the United States, and India. GARNet's members represent a variety of disciplines, including psychology, sociology, anthropology, sport and leisure sciences, public health, epidemiology, nursing, gerontology, human development, medical sciences, public policy, and human geography.

The focus of GARNet's work is a collaborative project to examine the social and environmental determinants of aging well. This project is using comparable data sets from the 12 member nations to develop an individual index of aging that is comparable within and across countries. This index will give policy makers a clearer and more comprehensive understanding of the factors that affect aging well. These findings can be applied in developing and evaluating social policies and services that will promote aging well across the life span.

The GARNet index measures items in each of the following five domains of aging well: physical health and functional status; mental/cognitive effectiveness; social support resources; daily life activity; and material security. A broad range of research and theoretical literature supports the importance of each domain.

**Physical health and functional status.** In cross-cultural studies of aging, good physical health and functional status have been associated with aging well (6). According to studies conducted by the MacArthur Foundation Network on Successful Aging, poor health in old age is largely attributable to the effects of multiple lifestyle choices and life conditions, such as physical inactivity, poor diet, and smoking, that increase the risk for disease or disability (11). Thus, maximizing physical health involves reducing these risk factors for disease and disability (11). Indicators for this determinant include health status, doctor and hospital visits, medication and supplement use, assistive device use, health promotion behavior, health risk behavior, and measures of functional independence associated with activities of daily living.

**Mental/cognitive effectiveness.** A critical domain of aging well is cognitive functioning, especially the cognitive effectiveness necessary to maintain the daily activities associated with overall health. Research by Baltes described two fundamentally different influences on the aging mind (8). The first, cognitive mechanics, is genetically endowed; the other, cognitive pragmatics, is culturally learned. Baltes speculates that older adults who lead effective lives use a framework of self-management and practical knowledge (cognitive pragmatics) to optimize gains and compensate for losses in cognitive mechanics. This view of cognitive efficacy fits well with descriptions of aging well. Indicators in this domain include measures of self-esteem, perceived control, resilience, and mental well-being.

**Social support resources.** A strong network of friends and family has long been recognized as an important contributor to good health in old age. Isolation or the lack of a strong social network places people at increased risk for poor health. Furthermore, a strong social support network can actually buffer or reduce some of the effects of aging (11). Indicators in this domain include measures of the availability and use of social support, network types, and other social resources.

**Daily life activity.** Identity in old age is influenced by the roles assumed in society. Because adult life involves cyclic and complex stages of personal development mediated by transitions, role disturbance and continuity are significant determinants of health in later life. For example, retirement potentially removes a lifelong source of identity and may create multiple points of disturbance to a person's well-being. An old age of idleness, without purposeful activity to help maintain identity and health, may well undermine aging well. The MacArthur Foundation studies in successful aging examined productive activity in a broad sense, including "any activity, paid or unpaid, that generates goods or services of economic value" (11). This approach allowed the researchers to examine a wide range of roles through which older adults can contribute to society. They concluded that three factors have a major influence on productive activity in older adults: 1) health and functional capacity, 2) social support network, and 3) personal characteristics. This domain includes indicators of engagement in formal and leisure activities, as well as other measures associated with daily time use.

**Material security.** Another important domain of aging well is material security (6). Having the material resources necessary to secure housing, sustenance, and access to needed services (e.g., medical care, social support) is fundamental to health and well-being in later life. In developed countries, retirement practices complicate the assurance of health and well-being in old age. For most of the past century, an ideal associated with retirement policies was to provide for the financial security of older Americans through social security and pensions; however, many older people, including women, minorities, unemployed people, and lower paid workers have not realized that ideal (6). Material security remains precarious for some older adults, such as very old people who have exhausted all of their resources. All individuals make decisions on the basis of providing for their basic needs (e.g., food, housing, safety) and for the basic needs of their families. When basic needs are unmet or compromised, personal health is compromised. The relationship between material security (conceived in broader terms than just income) and health is fundamental to aging well. The indicators in this domain include measures of access to shelter, food, services, safety, and economic resources, which are essential for health promotion and protection in all cultures worldwide.

These five domains are each individually well documented in the research literature. What remains to be determined, however, is how they operate interactively to circumscribe an individual's success at aging well. This is the question that GARNet's research is seeking to answer. By providing a more robust and universal understanding of the aging experience that goes beyond the medical perspective, the answer to this question could help us build a stronger foundation for health promotion and disease prevention initiatives.

Public health research and policy initiatives should embrace new paradigms that move health beyond the medical model toward a larger, more encompassing framework. The generation and testing of innovative frameworks that provide explanations of aging well are imperative for containing and reversing the impact of chronic disease and disability in old age. The aging of the population will only intensify this imperative. GARNet's findings, as well as those of others working in this area, are intended to lead us closer to a world where age is celebrated and aging well is a possibility for all older people.
